# Case of Retention of Fœtal Bones in the Uterus during Four Years

**Published:** 1847-01

**Authors:** James B. Ellis

**Affiliations:** Ripley, Miss.


*Case of Retention of Fœtal Bones in the Uterus during four years.* Communicated in a letter to the Editor, by JAMES B. ELLIS, M.D., of Ripley, Miss.

On the 29th of January, 1846, I was called by Dr. James Thompson, to see Mrs. Mary Ann Fields, about 25 miles distant from this place, aged 27 years, who gave me the following account of her case:

In Cass county, Georgia, early in April, 1842, she was confined at full time in her first pregnancy; her labour was very lingering, and the physician she sent for advised her to trust to the powers of nature, and left her. Her pains soon after ceased. At the time, she was unable to rise from her bed, and she therefore sent for two other medical gentlemen, and requested them to assist her. They told her that her pains would very probably return again, and all would terminate well. Her pains, however, did not return, and in about four months after, the decomposed flesh of the child commenced passing off rapidly, per vaginam ; she said the fetor was insupportable to her friends, so much so that visiters could scarcely come into her room. In about six months from her confinement to bed, the discharges ceased, and her health began to improve rapidly.

From this time her menstrual discharges returned, and continued regular in time, quantity and consistence, for more than three years. During this period there was a tumour in the epigastrium, that always gave a dull pain on pressure. Her disposition was cheerful, and continued as good as usual during this time. She consulted every medical gentleman that she could conveniently, but none agreed with her that the bones of the fœtus remained in the uterus. One expressed it as his opinion that the case was one of extra uterine conception. This, however, could hardly have been so, for she says the putrid flesh passed away rapidly per vaginam. About the middle of the summer of 1845 she was taken with typhoid fever, a disease that prevailed very generally in this section of country at that time. During the existence of her illness, there was considerable pain developed in the tumour spoken of, and she expressed to her attending physician her confident belief that the bones of her child were still in the uterus, and requested him to endeavour to extract them, but she said he only laughed at her and told her such a thing was impossible. After the abatement of her fever, her physician discontinued his visits, but she was still unable to walk from the violence of the pains in the uterus. During the succeeding winter, my friend Dr. James Thompson was called to see her. He found the tumour very large and painful, and the os uteri hard, and apparently sealed, and her general health very bad. He commenced treating her case on general principles, giving her tonics, alterants, anodynes, hip baths, etc. During the time I was occasionally consulted by him with reference to her case. A few days before I was first called to see her, she passed several very hard rough substances per rectum, which on examination were found to be bones. In a day or two she passed 25 or 30 bones, mostly small. At my request, Dr. Black accompanied me to see her, and on examination per rectum, I could distinctly feel a large fistulous opening from the rectum to the uterus, and could tell from her discharges that ulceration was going on rapidly. The ends of some of the long bones passed into the rectum and prevented a free discharge from her bowels, and at every attempt at defecation the bones were borne down against the raw edge of the fistula, which gave her excruciating pain. We had no instruments with us at the time for extracting the bones, having gone as much for curiosity as any prospect of doing her any good. We mustered up such rude instruments however as we could command on the spot, and requested Dr. Laird to assist us. With these we dilated the rectum, and with a pair of long curved forceps extracted the projecting bones. The operation was so very painful, the whole mucous membrane of the rectum being ulcerated, that she utterly refused to suffer any attempts at further efforts. She said she knew she would die, and would not suffer any pain that she could avoid.

We recommended Dr. Thompson to use anodyne mucilaginous enemas, pulv. charcoal, as a laxative, warm astringent hip baths, and such tonics as she could bear. This treatment she submitted to, although sometimes reluctantly. She continued to pass off bones more or less every day, and sometimes entertained hopes of recovery. At her death, (about the 20th of March, 1846,) Dr. Thompson thought she had discharged nearly all the bones, only a few of the cranium remaining. The fistulous opening was very large at the time of her death. A post mortem examination, I regret to state, could not be obtained. The statements made by Mrs. Fields, of her symptoms and sufferings, are all confirmed by her husband, and I have no doubt of their correctness. Some of the bones are in my possession now, which I will exhibit to you next winter, when I hope to have the pleasure of listening to your lectures again.

I have the honor to be, very respectfully,
Your friend and obedient servant,
JAMES B. ELLIS.

